# Non-Cannabinoid Metabolites of *Cannabis sativa* L. with Therapeutic Potential

**DOI:** 10.3390/plants10020400

**Published:** 2021-02-20

**Authors:** Henry Lowe, Blair Steele, Joseph Bryant, Ngeh Toyang, Wilfred Ngwa

**Affiliations:** 1 Biotech R & D Institute, University of the West Indies, Mona 99999, Jamaica; lowebiotech@gmail.com (H.L.); jbryant@ihv.umaryland.edu (J.B.); 2 Vilotos Pharmaceuticals Inc., Baltimore, MD 21202, USA; ngeh.toyang@flavocure.com; 3 Flavocure Biotech Inc., Baltimore, MD 21202, USA; 4 Institute of Human Virology (IHV), University of Maryland School of Medicine, Baltimore, MD 21202, USA; 5 Brigham and Women’s Hospital, Dana-Farber Cancer Institute, Harvard Medical School, Boston, MA 02215, USA; wngwa@bwh.harvard.edu

**Keywords:** cannabinoids, Δ^9^-tetrahydrocannabinol, cannabidiol, non-cannabinoids, flavonoids, terpenes, secondary metabolites

## Abstract

The cannabis plant (*Cannabis sativa* L.) produces an estimated 545 chemical compounds of different biogenetic classes. In addition to economic value, many of these phytochemicals have medicinal and physiological activity. The plant is most popularly known for its two most-prominent and most-studied secondary metabolites—Δ^9^-tetrahydrocannabinol (Δ^9^-THC) and cannabidiol (CBD). Both Δ^9^-THC and CBD have a wide therapeutic window across many ailments and form part of a class of secondary metabolites called cannabinoids—of which approximately over 104 exist. This review will focus on non-cannabinoid metabolites of *Cannabis sativa* that also have therapeutic potential, some of which share medicinal properties similar to those of cannabinoids. The most notable of these non-cannabinoid phytochemicals are flavonoids and terpenes. We will also discuss future directions in cannabis research and development of cannabis-based pharmaceuticals. Caflanone, a flavonoid molecule with selective activity against the human viruses including the coronavirus OC43 (HCov-OC43) that is responsible for COVID-19, and certain cancers, is one of the most promising non-cannabinoid molecules that is being advanced into clinical trials. As validated by thousands of years of the use of cannabis for medicinal purposes, vast anecdotal evidence abounds on the medicinal benefits of the plant. These benefits are attributed to the many phytochemicals in this plant, including non-cannabinoids. The most promising non-cannabinoids with potential to alleviate global disease burdens are discussed.

## 1. Introduction

A central Asian site of domestication of the cannabis plant is often cited [1]. The plant’s medicinal value was first recorded in the Pen Ts’ao Ching, the Chinese pharmacopoeia—the world’s oldest, compiled in the Han dynasty, first or second century A.D. [1]. The cannabis plant, its resin, and some derivatives of its compounds have been used in traditional Eastern medicine. Uses include, for recreation, due to its hallucinogenic/hypnotic effects; for religious celebrations and meditative purposes; industrially for fibers, textiles and ropes; and medicinally as an analgesic anticonvulsant, antidiarrheal, sedative, relaxant, anxiolytic, antibacterial, and antioxidant, and as treatment for tetanus, epilepsy and delirium tremens.

The cannabis plant produces an estimated 545 [2] chemical compounds. The main biogenetic classes of compounds produced are shown in Table 1 below. These include primary metabolites, such as amino acids, fatty acids, vitamins, sugars, and proteins, and secondary metabolites produced in trichomes, the cell’s factory. Primary metabolites provide nutritional value. Secondary metabolites also provide nutritional value and include approximately 120 terpenoids (61 monoterpenes, 52 sesquiterpenoids, and 5 triterpenoids [2], over 26 flavonoids [2], lignans, stilbenes and derivatives such as dihydro-resveratrol (3,5,4’-trihydroxybibenzyl) [3], cannabinaceous alkaloids such as cannabisativine and anhydrocannabisativine [4], and 20 steroids [2]), dihydrophenanthrenes, glycoproteins, and dibenzyls [5,6]. In addition to sharing analgesic, antimicrobial anticancer, anti-inflammatory, and neuroprotective properties, a majority also share antioxidant capabilities [7]. 

During many pathological conditions and diseases processes such as age-related inflammatory and autoimmune diseases, asthma, atherosclerosis, cancer, chronic obstructive pulmonary disease, hypertension, ischemia/perfusion, diabetes, HIV and dementia [8], the cells of the body accumulate high levels of toxic reactive oxygen species (R.O.S.)/free radicals in comparison to antioxidants [9]. This imbalance can cause significant damage to cell structures [9]. Antioxidants mitigate excessively produced reactive oxygen species within the cells, and in doing so, reduce the oxidative stress in cells [10]. Antioxidants may therefore have therapeutic applicability against many human diseases including but not limited to cancer, viral infections, cardiovascular diseases and inflammatory diseases [10]. Natural antioxidants may also be revered due to their safer side effect profile compared to synthetic antioxidants used in preventative medicine and in the food industry [10].

**Table 1:** The main biogenetic classes of compounds produced by *Cannabis sativa* L. [11].

	Main Chemical Classes	Number of Compounds	Reference
1	Terpenes	120	[11]
2	Cannabinoids	66	[11]
3	Hydrocarbons	50	[11]
4	Sugars and related compounds	34	[11]
5	Nitrogenous compounds	27	[11]
6	Non-cannabinoid phenols	25	[11]
7	Fatty acids	22	[11]
8	Simple acids	21	[11]
9	Flavonoids	21	[11]

**Figure 1:**
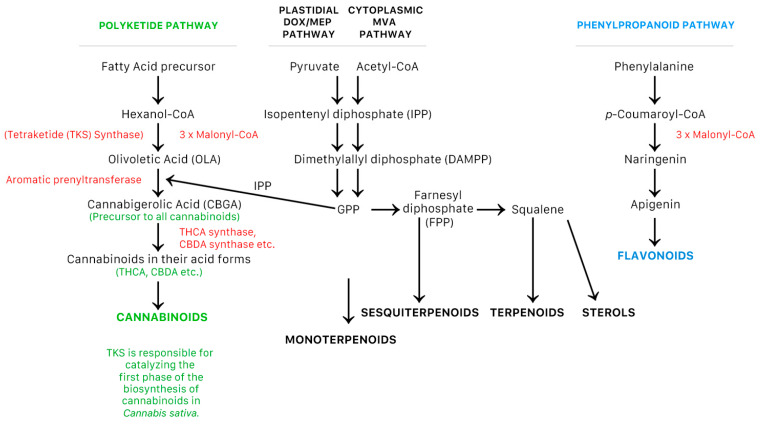
Synthesis of cannabinoids and non-cannabinoids produced by *Cannabis sativa* L. [2].

The biosynthesis of the non-cannabinoids in cannabis is driven by pyruvate/acetyl-CoA for the terpenes via the plastidial deoxyxylulose phosphate/methyl-erythritol phosphate (DOXP/MEP) and cytoplasmic MVA pathways [2], and *p*-coumaril-CoA via the phenylpropanoid pathway [2] for flavonoids [2]. The synthesis of cannabinoids is via the precursor molecule olivetolic acid (OLA) and the polyketide pathway, and another precursor molecule, olivetolic acid (GPP) and the plastidial deoxyxylulose phosphate/methyl-erythritol phosphate (DOXP/MEP) pathway [2]. These processes are simplified in Figure 1 below.

In addition to having unique organoleptic properties [12], terpenes provide a wide range of therapeutic benefits to humans, secondary metabolites, particularly terpenes and flavonoids, play a primary role in a plant’s defenses against hostile environments, herbivores and phytophagous insects.

The identified non-cannabinoid metabolites of cannabis are discussed in more detail in the ensuing sections.

## 2. Non-Cannabinoid Compounds

Cannabinoid and non-cannabinoid phytochemicals possess bioactive and protective properties that are beneficial to human health. In addition to cannabinoids, the cannabis plant also produces hundreds of non-cannabinoid secondary metabolites including approximately 120 terpenoids (61 monoterpenes, 52 sesquiterpenoids, and 5 triterpenoids [2], essential oils, over 26 flavonoids [2] lignans, stilbenoid derivatives, alkaloids, amino acids, spiroindans, polyphenols, 20 steroids [2]), dihydrophenanthrenes, glycoproteins (such as galactose, glucose, mannose and xylose), and dibenzyls [5,6]. This list also includes a-cannabispiranol, chrysoeriol, 6-prenylapigenin, cannflavin A and b-acetyl cannabispiranol [13].

Flavonoids and terpenoids in particular have a wide therapeutic window, including but not limited to cancer, inflammation, excessive reactive oxygen species in cells, and enhancement of neuroprotection. In addition to the phytochemicals mentioned below, a 2008 study by Radwan and colleagues identified and isolated six new non-cannabinoid phytochemicals from a high potency *Cannabis sativa* L. strain. These were 5-acetoxy-6-geranyl-3-n-pentyl-1,4-benzoquinone, 4,5-dihydroxy-2,3,6-trimethoxy-9,10-dihydrophenanthrene, 4,7-dimethoxy-1,2,5-trihydroxyphenanthrene, cannflavin C, cb-sitosteryl-3-O-b-D-glucopyranoside-20-O-palmitate, and 4-hydroxy-2,3,6,7-tetramethoxy-9,10-dihydrophenanthrene [13]. The review below focuses on non-cannabinoid phytochemicals with therapeutic potential.

### 2.1. Terpenes and Their Derivatives, Terpenoids

**Figure 2:**
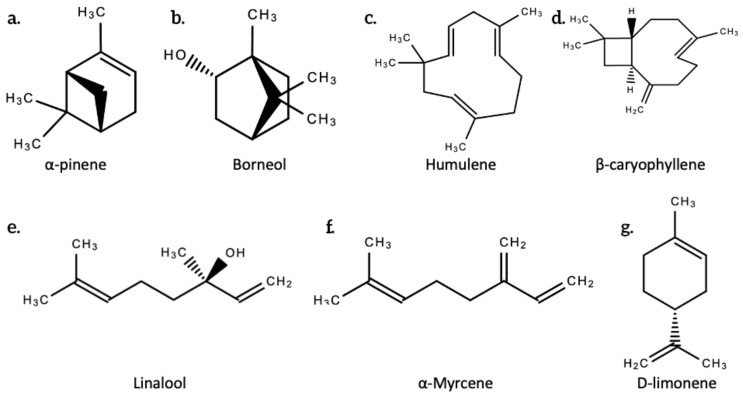
The chemical structures of some terpenoids produced by *Cannabis sativa* L. (**a**.) α-pinene; (**b**.) Borneol; (**c**.) Humulene; (**d**.) β-caryophyllene; (**e**.) Linalool; (**f**.) α-Myrcene; (**g**.) D-linonene.

The word “terpene” was devised in 1866 by August Kekulé, a German organic chemist. Terpenes are hydrocarbons and are made up of isoprene units (5-carbon building blocks) [14], while terpenoids are an oxidized and denatured form of terpenes that differ in that they contain an additional functional group with oxygen [14]. They are not the same, despite being used synonymously. This oxidation occurs during the drying and curing processes when the plant is exposed to open air. Terpenes are typically classified by the number of isoprene units in the molecule. Isoterpene is the only hemiterpenes. Hemiterpenes and hemiterpenoids such as prenol and isovaleric acid only have a single isoprene unit. Monoterpenes and monoterpenoids such as pinene (most common terpene produced across plant species), limonene, myrcene, geraniol, and terpineol have two isoprene units. Sesquiterpenes and sesquiterpenoids such as humulene and farnesol have three isoprene units. Triterpenes, such as squalene—the precursors to all steroids [14]—have six isoprene units. Sesquiterpenes and tetraterpenes have seven and eight isoprene units, respectively. Polyterpenes and norisoprenoids have multiple isoprene units in their molecule. Figure 2 below shows the structures of some terpenoids.

Figure 1 above shows the biosynthetic pathway of terpenes and terpenoids, which are synthesized from an isoprenoid precursor Isopentenyl pyrophosphate (IPP). This is achieved via the plastidial deoxyxylulose phosphate/methyl-erythritol phosphate (DOXP/MEP) pathway (monoterpenoids), and the cytoplasmic mevalonate (MVA) pathway (sesquiterpenoids, triterpenoids, and sterols) [2]. Along with cannabinoids, terpenes are considered a main physiological marker of secondary metabolites [15,16].

Terpenes are a large and diverse class of aromatic compounds responsible for the unique flavors and scents of many herbs and plants and phenotypic variation across plant species. Over 20,000 terpenes exist across plant species [17], and over 150 alone in the cannabis plant [18]. This makes terpenes the largest classification of phytochemicals (Andre, Hausman, Guerriero, 2016). Their primary role in plants vary but, in some plants, may attract pollinators, and in others, deter herbivores and inhibit microbial growth.

The most common terpenes in cannabis include limonene, α-pinene, β-pinene, humulene, β-caryophyllene, linalool and myrcene [2]. Other common terpenes in cannabis include bisabolol, borneol, camphene, geraniol, ocimene, terpineol, and valencene. These fall into the monoterpenoids and sesquiterpenoids categories. Refer to Table 2 below for examples of mono- and sesquiterpenoids profiled in a 2020 study by Jin and colleagues that profiled secondary metabolites in cannabis inflorescences, leaves, stem bars, and roots for medicinal purposes [2].

Terpenes provide aromatherapeutic benefits to humans including stress, anxiety, and depression relief, decongestion, and a general pharmacologically synergistic effect in combination with cannabinoids and flavonoids. As a result, terpenes are frequently used in cosmeceuticals, perfumes and beverage flavoring.

Terpenes and terpenoids are highly concentrated in the essential oils extracted from the cannabis plant, and are also responsible for the therapeutic benefits that essential oils provide. A study by Piccaglia et al., 2016 explored the biological properties of some terpenes making up said essential oils extracted from the cannabis plant (Table 3) by distillation and characterized by gas chromatography–mass spectrometry (GC–MS). Table 3 also shows the biological properties of other common terpenes found in cannabis.

The Medical Cannabis Network also reports that a current study is being undertaken by researchers at the Israel Institute of Technology investigating the therapeutic efficacy of a cannabis terpene inhalant formulation in suppressing the immune system response against COVID-19.

### 2.2. Phenolic Compounds in Cannabis sativa

**Figure 3:**
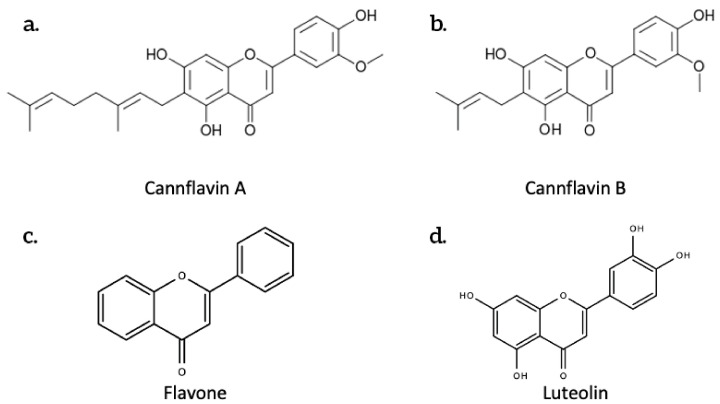
The chemical structures of some flavonoids produced by *Cannabis sativa* L. (**a**.) Cannflavin A; (**b**.) Cannflavin B; (**c**.) Flavone; (**d**.) Luteolin.

Polyphenols, also known as phenylpropanoids, are a class of over 10,000 chemical compounds that have phenyl groups/rings (C_6_H_5_). A phenyl ring consists of a 6-carbon structure formed on a hexagonal plane. Five of these carbon atoms are each individually bonded to hydrogen atoms (Figure 3a).

A 2020 study by Izzo and colleagues analyzed some phenolic compounds in *Cannabis sativa* L. inflorescences using ultra-high-performance liquid chromatography-quadruple-orbitrap high-resolution mass spectrometry (UHPLC-Q-Orbitrap HRMS). Table 4 below lists some of the phenolic compounds investigated. Cannabisin A, B and C were the predominant lignanamides identified [34].

### 2.3. Flavonoids

Flavonoids are a large family of polyphenolic plant compounds that naturally occur in fruits, vegetables, chocolate, and beverages such as wine and tea.

There are six classes of flavonoids including anthocyanidins, flavan-3-ols, flavonols, flavanones, flavones, and isoflavones. Chemically, flavonoids have the general structure of a 15-carbon skeleton, which consists of two phenyl rings (A and B), and a heterocyclic ring. This carbon structure can be abbreviated C6-C3-C6. Figure 3 below shows the structures of some flavonoids.

Flavonoids are found in a wide range of plants and may act as physiological regulators, cell-cycle inhibitors and/or chemical messengers. They are also responsible for plant pigmentation (flower coloration to attract pollinator animals/insects), UV filtration/protection, and symbiotic nitrogen fixation.

Flavonoids provide color, flavor and aroma. These class of compounds also have anticancer, antioxidant, antithrombogenic, antidiabetic and neuroprotective activities via modulation of a number of cell-signaling cascades.

A 2016 study by Bertoia and colleagues explored the relationship between dietary flavonoid intake (of flavonols, flavones, flavanones, flavan-3-ols, anthocyanins, and flavonoid polymers) and weight change at 4-year intervals between 1986 and 2011 of 124,086 men and women. In conclusion of this study, intake of foods rich in flavonols, flavan-3-ols, anthocyanins, and flavonoid polymers, inversely related to weight gain. Anthocyanidins had the greatest negative correlation with weight maintenance [35]. See Table 5 and Table 6 below for some biological properties of some common classes of flavonoids.

Over 20 different flavonoids have been identified in cannabis, most of which fall into the flavonol and flavone group [2]. One such flavonoid is cannflavin A, which is considered to be 30 times more effective than Aspirin at inhibiting prostaglandin E2, a significant modulator of inflammation [37]. Cannflavin B is also an inhibitor of prostaglandin E2 [38].

**Figure 4:**
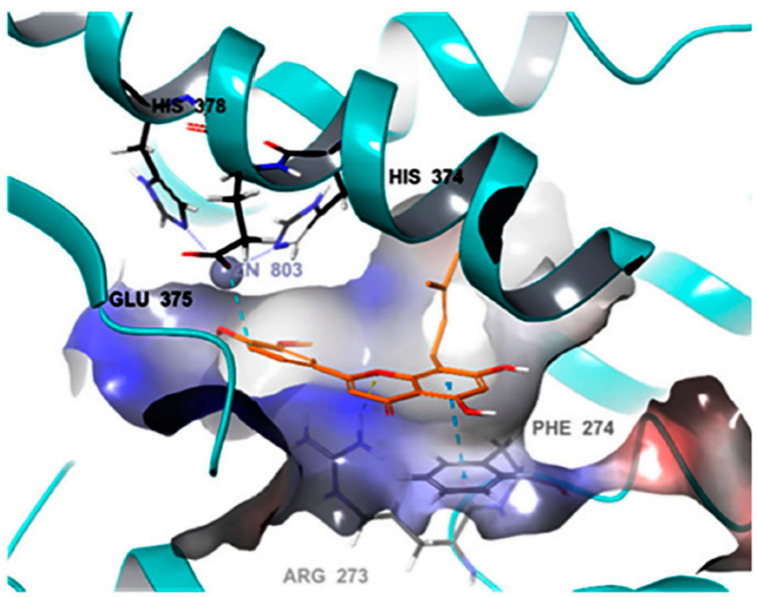
The main protein interactions of caflanone [39]. Reproduced with permission from Wilfred Ngwa, Potential of Flavonoid-Inspired Phytomedicines against COVID-19, published by *Molecules* Open-Access Journal, 11 June 2020 [39].

**Figure 5:**
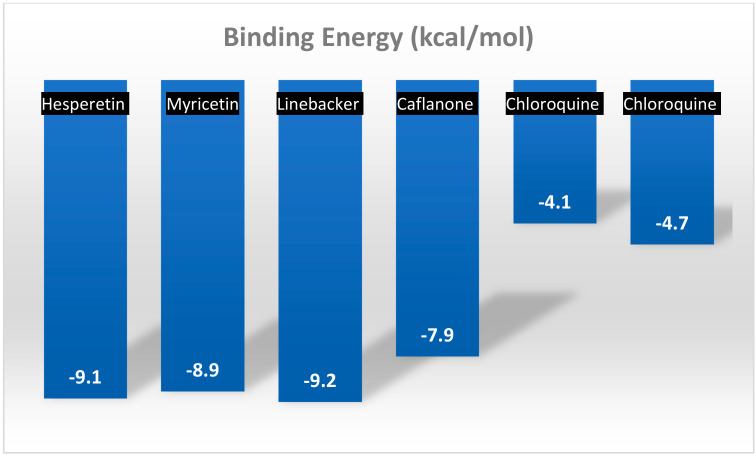
A comparison of the docking/binding studies results between the phytoantiviral flavonoids hesperetin, myricetin, linebacker, and caflanone [39].

Another major flavonoid derived from cannabis is caflanone. In recent studies, caflanone demonstrated activity against the human coronavirus OC43 (HCoV-OC43) also known as the severe acute respiratory syndrome coronavirus 2 (SARS-CoV-2). HCoV-OC43 belongs to clade b of the genus *Betacoronavirus* [39]. In vitro, caflanone inhibited HCoV-OC43 with an EC50 of 0.42 µM [39]. In silico studies showed that caflanone may act by inhibiting the angiotensin-converting enzyme 2 (ACE2) receptor found in the lung and respiratory tract, and used by the virus during cell entry and infection. Caflanone was also shown to have strong binding affinity to two of the proteases (PLpro and 3CLpro) are vital to the replication of SARS-CoV-2 in humans, thereby inhibiting viral entry to and/or replication within human cell [39]. Figure 4 shows the main interactions of caflanone with the proteins ACE2 (catalytic site/zinc metallopeptidase domain), Glu375, Glu402, ZN803, HIS374, Phe274 and Arg 273 [39]. The docking/binding studies results below show that the phytoantiviral flavonoids (Hesperetin, myricetin, linebacker, and caflanone) could bind equally or more effectively than chloroquine (CLQ), another investigated drug for SARS-CoV-2 [39] (Figure 5).

**Table 6:** More therapeutic benefits of flavonoids.

Therapeutic Window/Benefits of Flavonoids	Patent Number
Flavonoid derivatives targeting kinases, sirtuins and oncogenic agents for the treatment of cancers.	[40,41]
Agent containing flavonoid derivatives for treating cancer and inflammation.	[42]
Therapeutic agents containing cannabis flavonoid derivative for ocular disorders.	[43]
Therapeutic agents containing cannabis flavonoid derivatives for the prevention and treatment of neurodegenerative disorders.	[44]
Pi 4-kinase inhibitor as a therapeutic for viral hepatitis, cancer, malaria. autoimmune disorders and inflammation, and a radiosensitizer and immunosuppressant.	[45]
Therapeutic antiviral agents containing cannabis cannabinoid derivatives.	[46]

### 2.4. Fatty Acids of Cannabis Seeds

**Figure 6:**
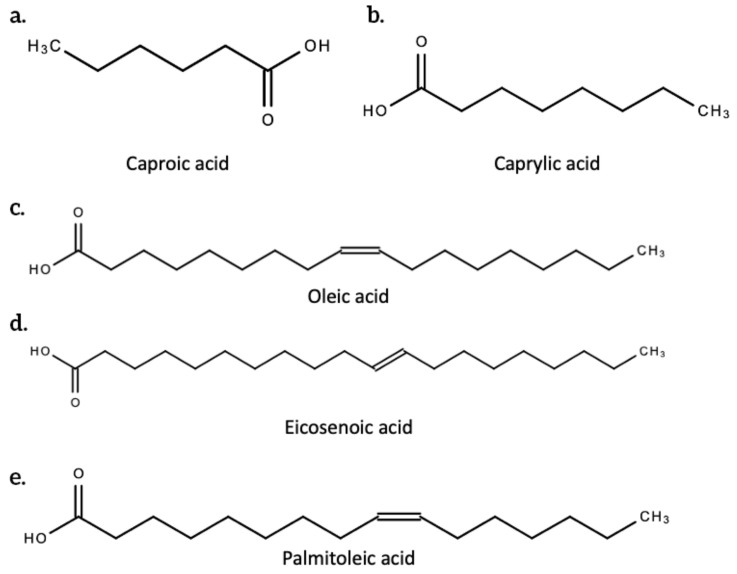
The structure of some fatty acids found in *C. sativa* L. [48]. (**a**.) Caproic acid; (**b**.) Caprylic acid; (**c**.) Oleic acid; (**d**.) Eicosenoic acid; (**e**.) Palmitoleic acid.

Fatty acids have high nutritional value. They are a class of molecules made up of a chain of carbon atoms bonded with hydrogen atoms, with a function carboxyl group (-COOH) attached to a terminal end. It is this functional group that participates in chemical reactions that allow fatty acids to perform their physiological roles. Figure 6 below shows the structure of some fatty acids produced by *C. sativa* L. These roles include providing insulation, storing and providing energy for cells in the absence of glucose, providing the precursor (cholesterol) for the production of hormones and intracellular membranes (estrogen, testosterone, vitamin D hormone, steroids, and prostaglandins), forming the building blocks of glycolipids and phospholipids which form the cell membrane and subcellular membrane, transporting fat soluble vitamins (A, D, E and K), and modifying proteins. Many of these fatty acids have nutritional and pharmaceutical potential [47].

A 1996 study by Ross and colleagues explored the composition of fatty acids present in the lipid matter of [commercial] cannabis seeds from different countries. Some fatty acids in commercial *Cannabis sativa* (seeds) include caproic acid, caprylic acid, myristic acid, palmitoleic acid, palmitic acid, margaric acid, oleic acid, linolenic acid, isolinolenic acid, linoleic acid, stearic acid, eicosenoic acid, arachidic acid, isoarachidic acid, and behenic acid [48].

It should also be noted that *C. sativa* L. seeds, particularly those of the hemp variety, typically have nutritional value. The seed is made up of approximately 25% high-quality protein and 35% fat [49]. Table 7 below lists some vitamins, minerals and macronutrients found in *C. sativa* L. seeds. In addition to significant concentrations of fatty acids (particularly omega-6 fatty acids), hemp oil extracted from *C. sativa* L. seeds also contain vitamin C [50,51]; thiamine [50,51]; riboflavin [50,51]; vitamin E [50,51]; minerals such as calcium [50], magnesium [50,51], potassium [50,51], phosphorus [50,51], iron [50,51], zinc [50,51], sodium [50,51], and copper [51]; and macronutrients such as fat [50], carbohydrates [50], fiber [50], and protein [50].

**Figure 7:**
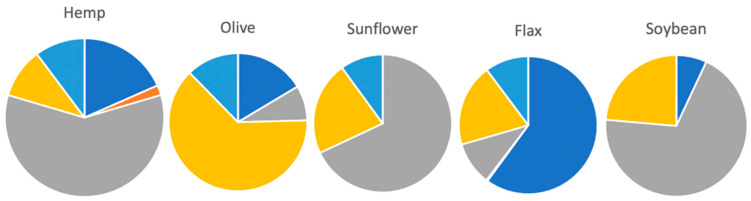
Comparison of the fatty acid composition of edible plant oils [49].

In comparison to other popular and nutritionally-rich edible oils such as canola, sunflower, pumpkin and soybean oils, only hemp oil provides the optimal ratio (3:1) of omega-6 fatty acid (linolic acid) and omega-3-fatty acids (alpha-linolenic acid), along with gamma-linolenic acid (GLA) and stearidonic acid (SDA) [49]. See Figure 7 for a comparison of the composition of edible plant oils.

### 2.5. Alkaloids in Cannabis

In 1876, Preobrajensky claimed to find nicotine in cannabis resin (hashish) from Uzbekistan in 1876 [52]. This was later rejected on the basis that cannabis users from that part of the world tend to mix the resin with tobacco before smoking. This made it likely that the presence of nicotine was due to the tobacco. Later in 1881, at the British Pharmaceutical Conference, Siebold and Bradbury reported on the isolation of the alkaloid cannabinine [53]. Two years later, in 1883, Hay isolated tetanocannabin, another biologically active alkaloid. It was so called because it produced strychnine-like convulsions in frogs [54]. In 1986, even Merck (of Darmstadt) began marketing and advertising a “cannabine alkaloid” product [55].

Alkaloids are a class of heterocyclical organic compounds that contain one or more nitrogen atoms. They may also contain an oxygen, sulfur, chlorine, bromine, or phosphorus atom attached to the molecule. They are most associated with plants, but are also produced by microorganisms and animals [56]. In plants, alkaloids are a form of chemical defense against herbivores. Many alkaloids are pharmacologically active. In fact, alkaloids make up about 60% of plant-derived drugs [57]. On the same tangent, dndogenous indole alkaloids have been confirmed in hemp [58].

Alkaloids have a variety of therapeutic applications as analgesics, antibacterial, anticancer, antiarrhythmic, antiasthma agents, antimalarials, anticholinergics, bronchodilatory, laxative, miotic, oxytocic, vasodilatory, psychotropic, and stimulating agents. This class of compounds include morphine, cocaine, nicotine, caffeine, quinine, ephedrine, among others.

Cannabinaceous alkaloids are alkaloids produced by *Cannabis sativa*. These include, but are not limited to, cannabisativine and anhydrocannabisativine [59].

A 1971 study by Klein and colleagues investigated the constituents of alkaloids mixtures extracted from cannabis plants and reported the isolated of four alkaloids, namely cannabimines A–D [60]. It was also noted that both cannabimine A and anhydrocannabisativine (isolated in 1976) share the same molecular formular (C21 H37 N3 O2) [59].

**Figure 8:**
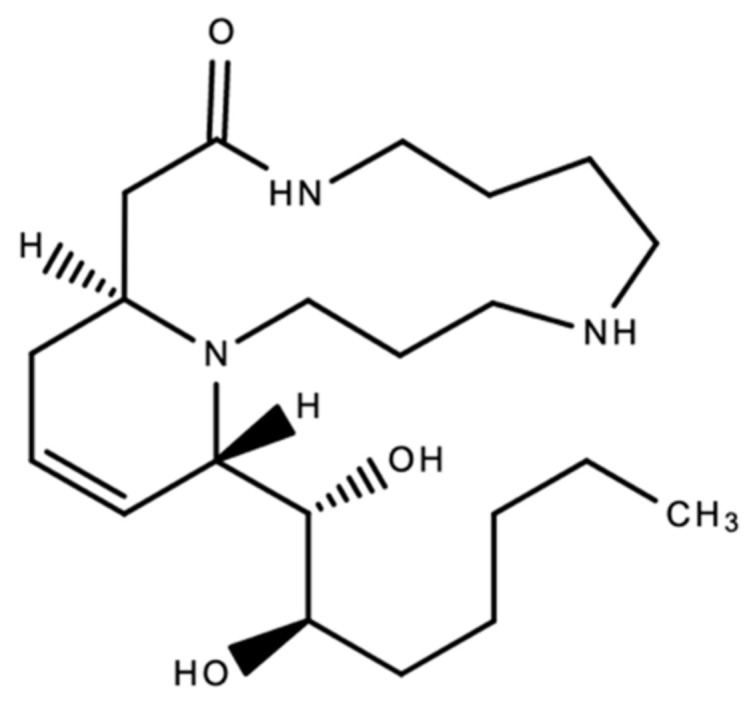
The chemical structure of cannabisativine, the first cannabinaceous alkaloid to be fully identified.

Cannabisativine was the first cannabinaceous alkaloid to be fully identified. It was isolated in 1975 in Mississippi from the roots of a Mexican variant of *Cannabis sativa* [61]. See Figure 8 below. These alkaloids demonstrated antiparasitic, antipyretic, antiemetic, antitumor, diuretic and analgesic properties [62]. In a 2004 study, Kuethe and Comins were able to achieve total asymmetric synthesis of cannabisativine with a high degree of stereo control [63].

### 2.6. Lignanamides and Phenolic Acids

Lignanamides are another example of naturally occurring, non-cannabinoid secondary metabolites with bioactivity. This class of phytochemicals produced in cannabis include Cannabisin A–G, and Grossamide [64].

These phytochemicals are powerful anticancer, antitumor, antiviral, antioxidant, antidiabetic, cardiovascular, cytotoxic, antineoplastic, anti-inflammatory, anti-obesity, analgesic, antihyperlipidemic and antihyperlipidemic agents [64], and they also inhibit acetylcholinesterase [65]. In this 2015 study by Yan and colleagues, four new lignanamides were also isolated from the hemp seed. These were Cannabisin M, Cannabisin N, Cannabisin O, and 3,3′-demethyl-heliotropamide [65]. Cannabisins are unique to the cannabis plant. It should also be noted that phenolic amides share these bioactive properties [65].

### 2.7. Amino Acids

**Figure 9:**
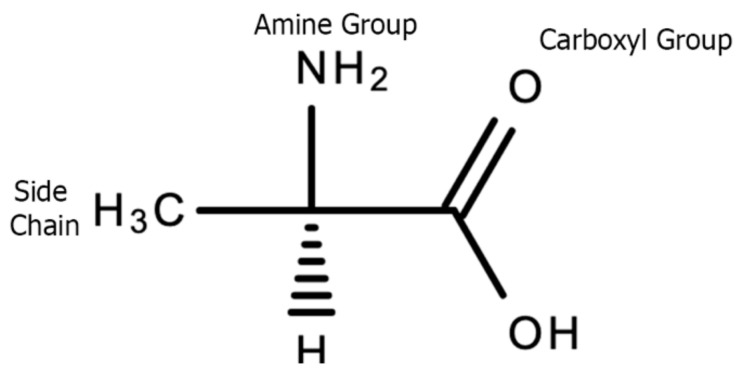
The basic structure of alanine, an amino acid.

Amino acids are the building blocks of proteins which are involved in multiple biological functions in the body including energy production, fat metabolism, muscle metabolism, immune system function, response and maintenance, the synthesis of enzymes, hormones and neurotransmitters, tissue growth, nutrient absorption, sleep–wake cycles, regulation of blood sugar levels, hemoglobin production, digestion, and sexual function [66]. The amino acid molecule consists of a central carbon atom, an amino group (-NH2), a function R group side chain that defines the chemical properties of that specific amino acid, and a carboxyl group (COOH). Refer to Figure 9 below for the chemical structure of an amino acid.

*Cannabis sativa* (hemp) seeds contain nine essential amino acids, namely include histidine, isoleucine, leucine, lysine, methionine, phenylalanine, threonine, and valine [67]. Essential amino acids are defined as those that cannot be synthesized in our bodies, but instead have to be obtained through food. In this same 2014 study by Audu, and colleagues, it is suggested that amino acids might be most concentrated within the leaves of the *Cannabis sativa* plant [67]. This is understandable because trichomes (the cell’s factory) are most concentrated in the leaves of plant. In one study aspartic acid, glutamic acid and arginine were the most prominent amino acids produced in hempseed [68]. In another study, hemp (*C. sativa* L.) isolates produced significantly higher levels of essential amino acid to total produced amino acids (with the exception of lysine), in comparison to a soy protein isolate [69].

Hempseed protein is unique within the plant kingdom because it contains the highest composition (65%) of the globulin edistin which make up many enzymes, antibodies, and hormones in the body [70].

### 2.8. Stilbenes and Stilbenoids

Stilbenes (and their derivatives) are naturally occurring polyphenolic phytochemicals that, aside from being a form of herbivore and disease resistance mechanisms for many lower and higher plants in the plant kingdom [71], have a wide range of biological activity and medicinal value to humans [72]. Stilbenoids are stilbene derivates that have been hydroxylated [73].

**Figure 10:**
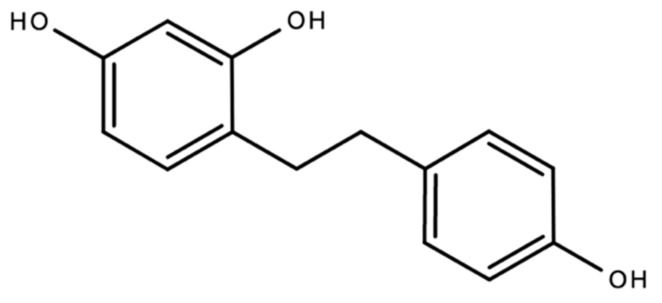
The chemical structure of dihydroresveratrol, a hydroxylated stilbene derivative.

A 2004 study by El-Feraly isolated, characterized and synthesized dihydro-resveratrol (3,5,4′-trihydroxybibenzyl), a metabolite derivative of the antioxidant, resveratrol, a dihydrostilbenoid from *Cannabis sativa* (Figure 10 above) [74]. A 2019 study by Giménez-Bastida and colleagues reported that dihydro-resveratrol and other metabolite derivatives of resveratrol induced senescence in breast cancer cells [75]. Studies on the antioxidant activity of dihydro-resveratrol are limited. A 2016 study by Tsang and colleagues report that dihydro-resveratrol demonstrated the ability to attenuate pancreatic oxidative damage and could possibly have therapeutic potential in the management of acute pancreatitis [3].

Nineteen stilbenoids unique to the cannabis plant were identified in a 1995 study by Ross and ElSohly [5]. These can be divided into three main structural classes: eleven spiroindans, eight dihydrostilbenes/bibenzyls, five phenanthrenes and prenylated, geranylated and glycosylated derivates [76]. Table 8 below is a list of these compounds.

**Figure 11:**
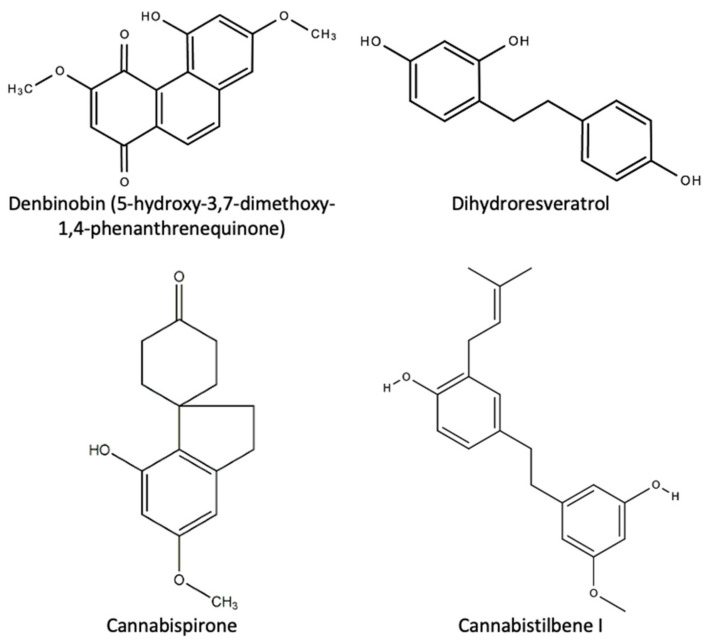
The chemical structures of some stilbenes and their derivates found in *C. sativa* L.

Denbinobin, first isolated in a 2008 study by Sànchez-Duffhues, is reported to have beneficial bioactive properties to human health as an anti-HIV, antioxidant, antitumor, and inhibitor of platelet aggregation [77]. The structure of denbinobin is shown in Figure 11 below.

## 3. Conclusions and Future Prospects

The cannabis plant produces an estimated 545 cannabinoid and non-cannabinoid phytochemicals of great economic and medicinal value [1]. These include some 104 cannabinoids (including CBD, Δ^9^-THC, and CBG), 120 terpenoids, over 26 flavonoids [1], lignans, phenolic amides, amino acids, nitrogenous compounds, steroids, fatty acids, alkaloids, stilbenes, vitamins, minerals and other phytochemicals. These phytochemicals are produced in virtually all areas of the plant (root, leaves, and stem). The aforementioned cannabinoids and non-cannabinoid secondary metabolites are produced primarily in the trichomes of the cannabis plant [78].

Cannabinoid and non-cannabinoid secondary metabolites have wide therapeutic application across many ailments including different types of cancers, diabetes, cardiovascular disorders, neurodegenerative disorders, inflammation-related diseases, and viral infections. Unlike THC, one of the most-studied cannabinoids, the majority of these phytochemicals are non-psychotropic. This means that they will not produce the psychoactive effects produced by THC, but will still have therapeutic benefit.

A majority of these phytochemicals require further in-depth characterization for their therapeutic efficacy and safety. There is also the need for a more rigorous standardization of cannabis cultivation practices so as to ensure consistent reproducibility of the profiles of secondary metabolites such as terpenes. The ability to reconstruct synthetic pathways, elucidate regulatory mechanism involved in the production of secondary metabolites, and establish genomic, metabolomic, transcriptomic maps/“fingerprints” of said secondary metabolites will allow drug manufacturers to produce more targeted drugs [79].

There is a need for more systematic botanical, physicochemical and chemical analyses of the cannabis plant. Further research is required to determine the efficacy, dosage standards, optimum extraction methods/solvents, cytotoxicity/hepatoxicity, pharmacokinetics, molecular mechanisms of action, phytoantiviral screening methods, and drug interactions for many phytochemicals in the cannabis plant.

## Figures and Tables

**Table 2:** Examples of mono- and sesquiterpenoids profiled in cannabis inflorescences, leaves, stem bars, and roots for medicinal purposes [2].

	Monoterpenoids		Sesquiterpenoids	
α-Pinene	Eucalyptol	Borneol	(-)-β-Elemene	Viridiflorol
Camphene	Ocimene	Terpinen-4-ol	β-Caryophyllene	(-)-Guaiol
Sabinene	γ-Terpinene	-Terpineol	Aromadendrene	(+)-Cedrol
(-)-β-Pinene	Sabinene Hydrate	(+)-Dihydrocarvone	Trans-β-Farnesene	β-Eudesmol
β-Myrcene	Terpinolene	Nerol	α-Humulene	α-Bisabolol
α-Phellandrene	Frenchone	Pulegone	Valencene	
Δ 3-Carene	Linalool	Carvone	Ledene	
α-Terpinene	Frenchol	Geraniol	Trans-Nerolidol	
p-Cymene	(-)-Isopulegol	Geranyl Acetate	Caryophyllene Oxide	
Limonene	Camphor		Globulol	

**Table 3:** The biological properties of some common terpenes in cannabis.

Terpene	Biological Property	References
Myrcene	Potent analgesic	[19]
	Antioxidant; neuroprotective; anti-inflammatory	[20]
	Anticonvulsant	[21]
1,8-cineole	Increases cerebral blood flow and enhances cortical activity	[22]
Limonene	inhibits many species of bacteria and fungi—repellant	[23]
	Anti-inflammatory; antioxidant; antiviral; antidiabetic; anticancer	[20]
	Antidepressant; anticonvulsant	[21]
α-Pinene	Antimicrobial; repellant	[23]
	Bronchodilator; anti-inflammatory,	
	Memory improvement/enhancement; acetylcholinesterase inhibitor	[21]
Linalool (Lavender scent)	Anxiolytic; anti-inflammatory; antimicrobial; anticancer; neuroprotective; antidepressant	[20]
	Anti-influenza	[24]
	Sedative; induces apoptosis in cancer cells	[21]
α-terpineol	Antimicrobial; repellant	[23]
	Anti-inflammatory	[25]
	Analgesic	[26]
	Nociception inhibition	[26]
	Anticonvulsant	[27]
	Antimicrobial	[21]
	Gastroprotective	[28]
Borneol	Antimicrobial; repellant	[23]
β- caryophyllene	Anti-inflammatory Analgesic	[20]
	Antispasmic in gut muscles	[21]
Humulene	Antiallergy; anticancer	[20]
Ocimene	Antifungal; antibacterial; antioxidant; antiviral; anti-inflammatory	[29,30,31,32,33]

**Table 4:** List of phenolic compounds in commercial *Cannabis sativa* L. [34].

Flavonols	Phenolic Amides	Flavones	Phenolic Acids
Catechin	N-trans-caffeoyltyramine	Cannflavin A	Hydroxycinnamic acids
Epicatechin	Flavonoids	Cannflavin B	Chlorogenic acid
Flavanone	Flavonol	Luteolin-7-O-glucoside	Caffeic acid
Naringenin	Rutin	Apigenin-7-O-glucoside	p-Coumaric acid
Lignanamides	Quercetin-3-glucoside	Luteolin	Ferulic acid
Cannabisin A	Kaempferol-3-O-glucoside	Apigenin	
Cannabisin B	Quercetin		
Cannabisin C	Kaempferol		

**Table 5:** The biological properties of some common classes of flavonoids.

Flavonoid	Biological Property	
Flavonols (e.g., quercetin and kaempferol)	Antioxidant; cardioprotective	[36]
Flavanones	Antioxidant; anticancer; anti-inflammatory	[36]
Isoflavonoids	Phytoestrogenic (mimic the hormone estrogen); hormone balance and metabolism	[36]
Anthocyanins (responsible for a plant’s unique colour)	Antioxidant and anti-inflammatory	[36]

**Table 7:** Examples of vitamins, minerals and macronutrients produced *by C. sativa* L. seeds [50].

Vitamins	Minerals	Macronutrients
C	Calcium	Fat
Thiamine	Iron	Carbohydrate
Riboflavin	Magnesium	Fiber
Niacin	Phosphorus	Protein
B-6	Potassium	kJ:2313
Folate	Sodium	
A	Zinc	
E		

**Table 8:** Some stilbenes and their derivatives found in *C. sativa* L.

Spiroindans	Eight Dihydrostilbenes/Bibenzyls	Phenanthrenes and Derivates
1. Cannabispirone 2. Cannabispirenone-A 3. Isocannabispirenone 4. Isocannabispiradienone 5. Cannabispirenone-B 6. β-cannabispiranol 7. α-cannabispiranol 8. Acetyl cannabispirol **Cyclohexane Spirans** 9. 5,7-dihydroxyindan-1-spiro-cyclohexane 10. 7-hydroxy-5-methoxyindan-1-spiro-ciclohexane 11. 5-hydroxy-7-methoxyindan-1-spiro-ciclohexane	**Prenylated** 3,4′-dihydroxy-5,3′-dimethoxy-5′ isoprenyl Canniprene Cannabistilbene I, Cannabistilbene IIa Cannabistilbene IIb **Non-Prenylated** 3,4′-dihydroxy-5-methoxy bibenzyl 3,3′-dihydroxy-5,4′-dimethoxy bibenzyl Dihydroresveratrol	9,10-dihydrophenanthrene cannithrene-1 cannithrene-2 4,7-dimethoxy-1,2,5-trihydroxyphenanthrene 4,5-dihydroxy-2,3,6-trimethoxy-9,10-dihydrophenanthrene 4-hydroxy-2,3,6,7-tetramethoxy-9,10-dihydrophenanthrene Denbinobin (5-hydroxy-3,7-dimethoxy-1,4-phenanthrenequinone)

## Data Availability

Data sharing is not applicable to this article. No new data were created or analyzed in this study.
